# Investigation of Roughness, Morphology, and Wettability Characteristics of Biopolymer Composite Coating on SS 316L for Biomedical Applications

**DOI:** 10.1155/2024/5568047

**Published:** 2024-01-17

**Authors:** Hanaa A. Al-Kaisy, Basma H. Al-Tamimi, Qahtan A. Hamad, Mayyadah S. Abed

**Affiliations:** Department of Materials Engineering, University of Technology, Baghdad, Iraq

## Abstract

This project aims to create a 316L stainless steel coated with a biocomposite based on chitosan for use in the biomedical industry. To completely coat the material, the dip-coating technique was used to apply plain chitosan, chitosan nanosilver, chitosan biotin, and chitosan-nanosilver-biotin in that order. This coating's surface morphology was investigated with field emission scanning electron microscopy (FESEM). Surface roughness, average size distribution, and 2D and 3D surface tomography were all investigated using scanning probe microscopy and atomic force microscopy (SPM and AFM). The Fourier transform infrared (FTIR) spectroscopy technique was used to quantify changes in functional groups. To evaluate the coated samples' wettability, contact angle measurements were also performed. The chitosan (CS) + nanosilver, CS + biotin, and CS + biotin + nanosilver-coated 316L stainless steel showed roughness values of about 8.68, 4.21, and 3.3 nm, respectively, compared with the neat chitosan coating, which exhibits 12 nm roughness, indicating a strong effect of biotin and nanosilver on surface topography whereas the coating layers were homogenous, measuring around 33 nm in thickness. For CS + nanosilver and CS + biotin, the average size of agglomerates was approximately 444 nm and 355 nm, respectively. The coatings showed adequate wettability for biomedical applications, were homogeneous, and had no cracks. Their contact angles were around 51–75 degrees. All of these results point to the composite coating's intriguing potential for use in biological applications.

## 1. Introduction

Biomaterials play a pivotal role in diverse fields such as biotechnology, medicine, dentistry, and bioreactor applications. Nevertheless, the biomaterials initially identified seven decades ago have become scarce, and the contemporary market is characterized by a diverse array of biomaterial types [1, 2]. These materials are versatile in many medical applications because of their vast variety of physical, chemical, structural, and mechanical characteristics [3, 4].

Medical implants, including knee implants and orthopedic screws and pins, commonly utilize stainless steel (grade 316L). Thus, there is a potential benefit in employing antibacterial coatings on 316 stainless steel to reduce the risk of postoperative infections [5–7].

Stainless-steel alloys confer distinct advantages in materials engineering, characterized by their cost-effectiveness, ready accessibility, superior machinability, biocompatibility, and exceptional mechanical strength [8]. Stainless steel (316L) has high biocompatibility because it has a stretching protective layer that is resistant to corrosion [9]. Dip coating, sol-gel coating, and electrophoretic deposition represent widely used methods for altering implant surfaces with biocomposites. The sensitivity of each technique to preparation parameters significantly influences the resulting coating properties [10].

Biopolymers found in nature, including chitosan and its derivatives, exhibit antibacterial, antimicrobial, biodegradable, biocompatible, and nontoxic properties [11]. Thus, they are widely used in the food and pharmaceutical industries [12]. Sanpo et al. [13] employed powder technology to create chitosan-copper composites for antibacterial coatings targeting E. coli. The findings demonstrated that the antimicrobial efficacy of all composite coating layers improved with an increase in the proportion of powders, attributable to the cold aerosol effect. Carneiro et al. [14] applied a coating of chitosan and the organic molecule 2-mercaptobenzothiazole (MBT) to aluminum alloy, resulting in the release of corrosion inhibitors. This chitosan-MBT combination served as an “intelligent” pigment, demonstrating suitability for high-performance applications. Ahmed et al. [15] applied electrophoresis techniques to deposit hydroxyapatite (HA)-zein on 316L stainless steel, aiming to improve its corrosion resistance. Analysis through scanning electron microscopy (SEM) and energy-dispersive X-ray spectroscopy (EDX) revealed robust adhesion and enhanced performance in biological environments, simulated through the utilization of simulated body fluid (SBF). Issa et al. [16] deposited a thin layer of poly methyl methacrylate (PMMA) reinforced with diverse bioceramics onto a stainless-steel substrate using electrostatic deposition. The resulting coating exhibited homogeneity, without any signs of cracking, and demonstrated satisfactory mechanical characteristics. Vafa et al. studied the effect of PVA concentration on chitosan/bioactive glass-coated AZ91D Mg Alloy [17]; they also used electrophoretic deposition of PVA/chitosan/bioactive glass on 316L SS [18].

Since nanosilver and biotin are biocompatible and have protective qualities, they are added to biopolymer composites to coat stainless steel 316L in biomedical applications [19, 20]. Nanosilver possesses antimicrobial properties [21], making it a valuable component that can contribute to lowering the risk of infection [19]. One vitamin that is essential for supporting tissue healing and cell development is biotin. It is especially beneficial for applications pertaining to wound healing because of this feature [19]. The composite coating's blend of biotin and nanosilver creates a material that, in addition to providing protection, actively encourages the body's natural healing processes [19, 20]. Additionally, electrochemically triggered macromolecular film buildup processes can be used to create a more durable coating with improved adhesion strength [21]. Finally, research has shown that HA-coated 316L SS specimens appear more biocompatible than uncoated specimens, making them suitable for use in biomedical applications [22].

Heidari et al. utilized other natural materials such as nano‐hydroxyapatite with zinc oxide [23], palladium [24], and magnetite [25] as scaffold for bone tissue engineering applications whereas others prepared hydrogel nanocomposites of chitosan/collagen/hydroxyapatite as a scaffold [26].

Plasma processing has been recently mentioned as a promising method to modify the surface of polymers for biomedical applications [27–30]. Plasma treatment can selectively modify the surface of polymers, introducing different chemical groups onto the surface and changing its properties [31, 32]. This can have a significant influence on cell-material interactions, making it an excellent candidate for polymeric materials used in biomedical applications [2].

Plasma polymerization is a deposition technique where a gaseous or liquid monomer is introduced in the plasma discharge and converted into a thin film coating on biomedical devices and products [33]. The biocompatibility of a polymer surface can also be changed by ion implantation, which promotes cell attachment and subsequent growth. The surface roughness of substrates made of modified polymers varies as a result of plasma treatment [29].

The biocompatibility of diverse polymer substrates treated with plasma to various cell lines reveals the need to change each substrate in a unique manner in order to achieve desired results. By using oxygen plasma etching, plasma processing can also be used to create super-hydrophilic or super-hydrophobic polymeric surfaces. For various biomedical purposes, nanoparticles may also be grafted onto the surface of polymers [34]. Overall, plasma processing has proved itself to be an effective, environment-friendly, and cost-efficient technology for modification of biomaterials surfaces for biomedical applications [29, 33].

Dip coating is a common method used to modify implant surfaces [35]. It is an effective way to improve the implant material and can be coupled with other coating techniques such as spin coating and spray coating [35, 36]. However, dip coating has drawbacks such as long processing time and low scratch resistance [37]. Bactericidal surfaces are also emerging as a 21^st^-century ultrahygienic surface treatment for implants [38].

Numerous attempts were made to develop the surface properties of 316L stainless steel (SS) which is used as an implant material by coating techniques. The reason to use the coating is to alter the surface properties of the substrate without influencing the properties of the whole implant. So, the aim of this work is to study the effect of using natural polymer-based (chitosan) as a coating on 316L stainless steel via the dip-coating method on its biological characteristics and then study the effect of adding biotin and nanosilver particles to the chitosan matrix as composite coatings on 316L stainless-steel samples. Then, characterization and investigation of surface properties would be performed to reveal the incorporation impact of biotin and nanosilver particles into the chitosan base. Surface morphology, roughness, agglomerates, average size, functional group modification, and wettability will be taken into account during this investigation.

## 2. Experimental Part

### 2.1. Materials

The 316L stainless-steel rod utilized in this study was procured from TISCO Industrial Co., China. Silicon carbide (SiC) grit abrasive paper of varying grades (80, 100, and 150) was obtained from Buehler company, United States. Deionized water utilized in the laboratory was prepared internally. Acetone, sourced from Fixtone Co., Jordan, was employed in the study. The nanosilver particles utilized were synthesized in a previous study through a green synthesis method. Chitosan (99.9%), biotin (99%), acetic acid (99.7%), and sodium hydroxide were supplied by Sigma Aldrich.

### 2.2. Preparation Procedure

Austenitic rods of 316L stainless steel, each measuring 1 mm in thickness and 20 mm in diameter, were employed as substrates. The surfaces of these substrates underwent grinding and polishing using silicon carbide (SiC) grit abrasive papers of grades 80, 100, and 150. Following this, the samples were cleansed with deionized water, degreased using acetone, and subsequently dried. Dip-coating technique was then employed to fabricate chitosan (CS)-based biocomposite coatings, as outlined in Table 1. Three specimens were produced for each composition, with the neat chitosan-coated 316L stainless steel serving as the control specimen.

The (90% chitosan + 10% biotin) and (90% chitosan + 5% nanosilver + 5% biotin) coating compositions were selected in this work for their potential to be used in active drug delivery systems[40, 41]. Biotin-tagged chitosan oligomers have been synthesized and investigated as suitable polymeric derivatives for the preparation of drug-loaded nanoparticles [42]. Chitosan is a versatile material that has been used in various technical, agricultural, and biomedical fields due to its biocompatibility, biodegradability and nontoxicity [43]. The combination of chitosan with biotin and nanosilver provides an effective platform for drug delivery due to the enhanced stability of the nanoparticles [40, 41]. The biotin-tagged chitosan oligomers can also be used to target specific cells or tissues for the drug delivery system [42].

CS powder was dissolved in diluted acetic acid by magnetic stirrer until a milky solution was made, and then the pH was measured. NaOH solution was used to raise the pH level to 6.0. Then, the CS solution was partially mixed with nanosilver, biotin, and nanosilver + biotin to prepare the biocomposite coating. The mixture was made using a stirrer 10 min and then sonicated for 15 min.

The cleaned 316L stainless-steel samples were dipped into the neat CS solution, CS + nanosilver, CS + biotin, and CS + nanosilver + biotin separately for 30 min.

Afterwards, samples were preserved in polyester cans after the coated specimens underwent another drying process at 27°C. Utilizing a scanning probe microscopy, AFM, FESEM, and a wettability test, the morphology of the coated specimens was described. The Fourier transform infrared (FTIR) method was used to assess each coating's chemical bonding.

### 2.3. Characterization Techniques

Fourier transform infrared (FTIR) spectroscopy instrument model (Bruker Optics Company, Germany) was utilized in order to indicate the molecular bond structure and functional groups of the neat chitosan coating and the chitosan matrix with nanosilver and biotin composite coatings while the structural homogeneity and nanoparticles distribution within matrix structure and the morphological characteristics of the as-prepared samples were accomplished using field emission scanning electron microscope (FESEM) model (TESCAN VEGA-SB, Belgium). Furthermore, the surface topography was studied by using the atomic force microscope AFM model (PHYWE/UK). Also, in order to determine the surface hydrophilicity of the as-prepared samples, the contact angle was measured by an optical contact angle meter “CAM 110-O4W.”

## 3. Results and Discussion

### 3.1. Fourier Transform Infrared (FTIR) Spectroscopy

**Figure 1:**
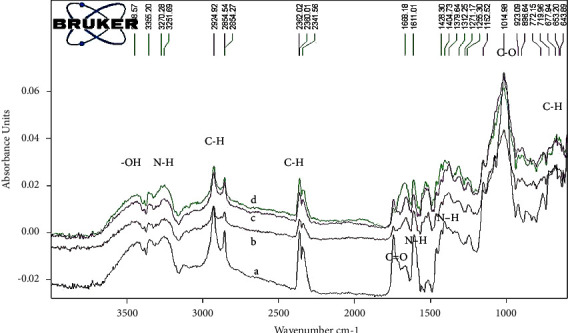
FTIR spectrum of (a) neat chitosan coating, (b) CS + nanosilver, (c) CS + biotin, and (d) CS + nanosilver + biotin composite coating layer on 316L SS substrate.

FTIR spectrophotometer was utilized to demonstrate the molecular bond structure and functional groups in the spectral range of 600–4000 cm^−1^ of the neat CS coating and the CS matrix with nanosilver and biotin composite coating.  Figure 1 shows the FTIR spectra of neat CS (curve a) and CS-composite coating (curves b, c, and d). The peaks at 3414.20, 3382.58, and 3347.52 cm^−1^ correspond to the stretching vibration of the –NH_2_ group. The curve a shows vibration at 3253.33 and 3352.08 cm^−1^ for N–H and –OH stretching vibrations, respectively, 2926.09 cm^−1^ for C–H stretch from alkyl groups, 1724.09 cm^−1^ for C=O stretching (amide I), 1604.98 cm^−1^ for N–H amine, and 1015.16 cm^−1^ for skeletal vibration of C–O [44].

The curves b and c illustrate the spectrum of the CS matrix with nanosilver and biotin, respectively. The presence of nanosilver reduced the intensity of CS bands, especially the large transmission band at 3257.27 and 3294.49 cm^−1^. The addition considerably decreased the band intensity due to the dual effect of metallic particles on the composition of the CS matrix. The addition of nanosilver and biotin in the CS-based composite layers decreased the intensity of the bands generated from amide II (NH) at 2924.03 and 2924.92 cm^−1^ of CS. The intensity of CH stretching (alkyl groups) increased at approximately 2326.34 and 2362.02 cm^−1^ for the CS composite matrix, 1742.86 and 1742.47 cm^−1^ for C=O stretching, and 1588.66 and 1400.45 cm^−1^ for N–H amine stretching frequency.

From the curves b and c, it can be noticed that a remarkable transformation occurred at the 1015.22 and 1015.54 cm^−1^ peaks (the number of waves increased) after the addition of silver and biotin to the CS coating. The band at 850 cm^−1^ appeared in the spectra of the neat CS coating and the CS/composite coating.

Generally, it can be observed the similar vibrational modes from FTIR spectra of the deposited coatings with minor differences attributed to the added Ag or biotin. The lack of a chemical reaction between the components indicated that the miscibility state had progressed [45], as well as the absence of byproducts that could induce harmful or allergic reactions within the human body. The curve d illustrates the spectrum of the CS matrix with nanosilver + biotin. The presence of nanosilver and biotin together reduced the intensity of the bands of chitosan, especially at a large transmission band of 3356.20 cm^−1^.

The stretched C–O bonds in the ester group correspond to the strong peaks at 1015.39 cm^−1^. C–H bending was represented by peaks at 920.20 and 842.91 cm^−1^. Similar FTIR spectra peaks were reported [46].

### 3.2. Morphological Analysis

**Figure 2:**
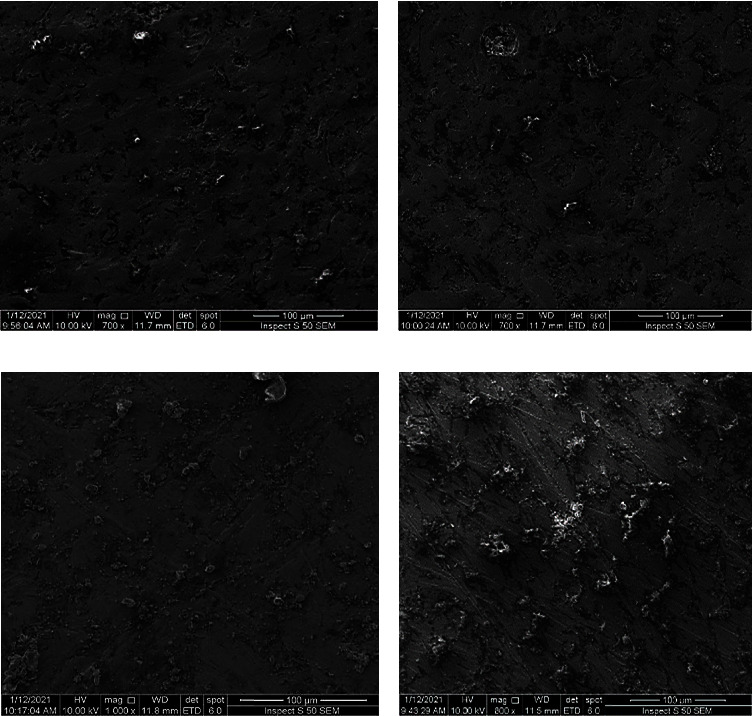
FESEM images of CS composite coated on 316LSS: (a) pure chitosan, (b) CS + nanosiliver, (c) CS + biotin, and (d) CS + nanosiliver + biotin.

FESEM was implemented to characterize the microstructures of the composite coating and the influence of the adding nanosilver and biotin on the surface morphology and roughness of the CS matrix.  Figure 2(a) shows a homogeneous porous film of neat CS coating.  Figure 2(b) shows the coated sample with CS + 5% nanosilver. The homogenous distribution of particles through the CS matrix can be observed. Similar observations were reported in [47, 48].

Figure 2(c) shows the microstructure of the chitosan matrix with 5% wt biotin; it can be noticed that biotin is responsible for the observed improvement in surface homogeneity and decrease in surface roughness.  Figure 2(d) shows the CS-nanosilver-biotin coated specimen; it reveals the homogenous distribution of the added materials to the natural polymer matrix. In general, the SEM images revealed the well-distributed clustered biotin or nanosilver immersed in the matrix. The simple porosity and absence of cracks are noted. This porosity confirms the presence of nanosilver and biotin within the matrix which promotes implant osseointegration.

**Figure 3:**
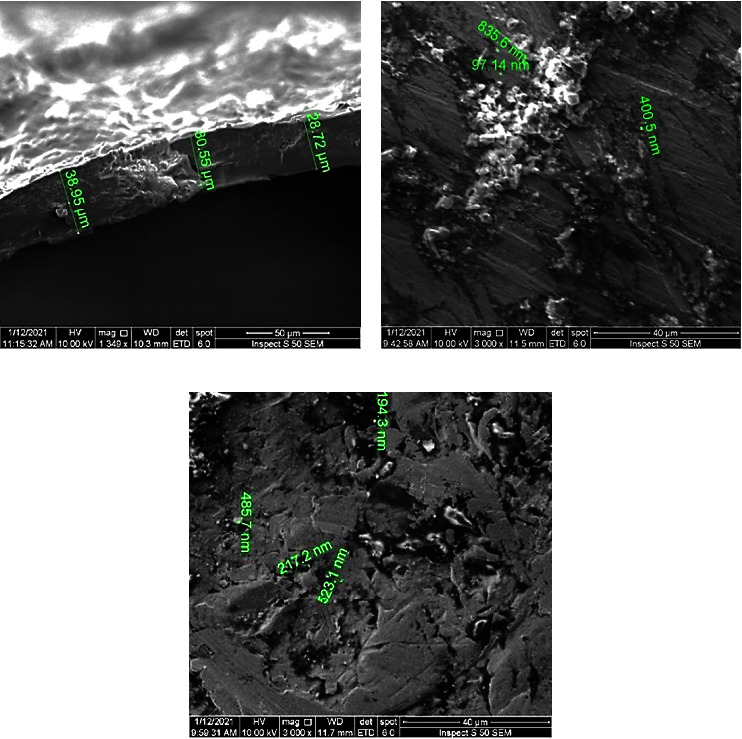
FESEM of the (a) coating thickness, (b) average size of CS + nanosilver agglomerate, and (c) average size of CS + biotin agglomerates.

The porous surface increased the implant's interfacial adhesion with the surrounding tissues, which improved the implant's mechanical stability. The composite coating thickness was obtained from SEM images (see Figure 3(a)), and it was about 33 nm. Moreover, the average size of agglomerates was about 444 nm for CS + nanosilver agglomerates and 355 nm for CS + biotin agglomerates (see Figures 3(b) and 3(c)).

### 3.3. Surface Roughness

**Figure 4:**
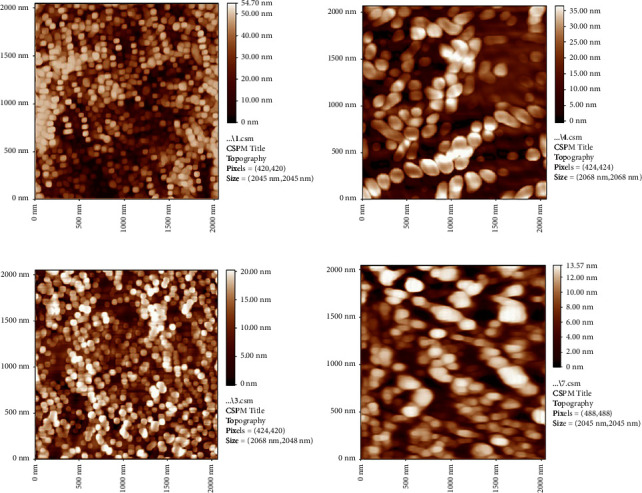
2D/AFM images of (a) neat chitosan, (b) chitosan + nanosilver, (c) chitosan + biotin, and (d) chitosan + nanosilver + biotin.

The 2D/AFM images of as-prepared samples (neat chitosan, chitosan + nanosilver, chitosan + biotin, and chitosan + nanosilver + biotin) are shown in Figures 4(a)–4(d), respectively. The figures give good indications about the surface characteristics of the prepared coating layers. The topographical characteristics can be obtained by different parameters that are used to quantify the root mean square (RMS) roughness of the surfaces. The RMS roughness values of the as-prepared samples surfaces of neat chitosan, chitosan + nanosilver, chitosan + biotin, and chitosan + nanosilver + biotin are 54.7 nm, 35 nm, 20 nm, and 13.57 nm, respectively. The RMS roughness values decreasing due to the addition of nanosilver and biotin particles compared to that of the neat chitosan are attributed to the association of these two materials and its effect on smoothing mechanism due to surface diffusion of these particles within the chitosan matrix.

**Figure 5:**
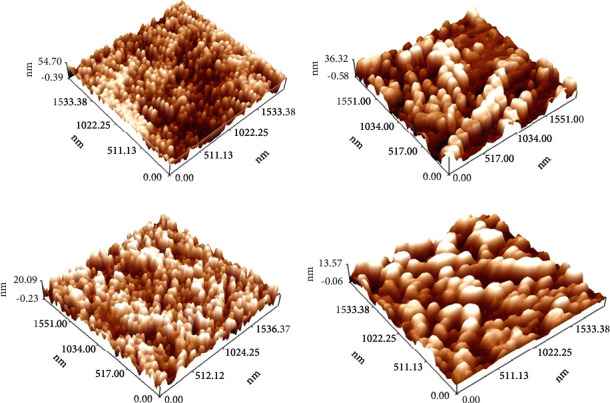
3D/AFM images of the (a) neat chitosan, (b) chitosan + nanosilver, (c) chitosan + biotin, and (d) chitosan + nanosilver + biotin.

**Figure 6:**
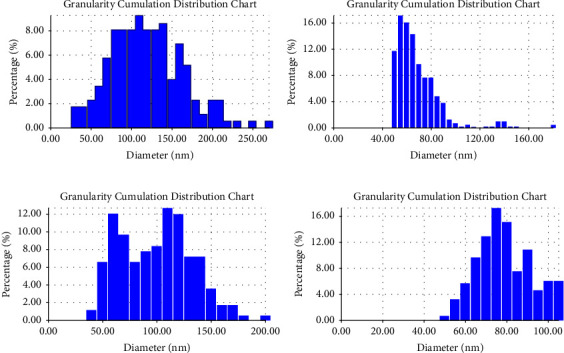
Cumulative distribution chart of the (a) neat chitosan, (b) chitosan + nanosilver, (c) chitosan + biotin, and (d) chitosan + nanosilver + biotin.

Figures 5(a)–5(d) refer to the 3D/AFM images of the as-prepared coating layers. These images agreed with the results in Figure 4 which confirm the well distribution and homogeneity of both nanosilver and biotin particles within the chitosan matrix and the smoothness of the surface roughness which has good influence on its characteristics for biomedical applications while the cumulative distribution charts of the as-prepared coating layers are shown in Figures 6(a)–6(d) for neat chitosan layer, chitosan + nanosilver layer, chitosan + biotin layer, and chitosan + nanosilver + biotin layer, respectively.

Table 2 summarizes the results of surface roughness and average diameter, which indicate that the addition of biotin and nanosilver particles presents extremely significant changes in surface coating properties, as shown in Figures 4–6, in which the gradual decrease in surface roughness is noticed due addition of nanosilver and biotin particles.

The composite-coated specimens were more homogeneous than the blended specimens (CS + nanosilver + biotin) because nanoparticles with biotin decreased the surface roughness and average diameter (Table 2) of the CS-based composite coating. The morphology of the composite coating is smooth, and the solid components had a uniform distribution, which reduced the roughness values (Ra) and average diameter [49].

The nanosilver precipitated at the surface of biotin within the chitosan matrix because of the large differences in particle size and surface charge of these particles, which decreased the roughness [50, 51]. The incorporation of metallic particles enhanced the overall characteristics, while varying the particle scale size helps to optimize the combinations and reduce surface roughness [50]. Chitosan is precipitated with polyanions and in alkaline solutions [51, 52].

The size and surface characteristics of chitosan microparticles or nanoparticles play an important role in their interaction with cells and cellular components. The particle size, stability, residual toxicity, and other properties of nanomaterials influence their cellular uptake and retention [52]. Therefore, the large differences in particle size and surface charge between biotin and nanosilver likely contributed to the precipitation of nanosilver at the surface of biotin within the chitosan matrix, resulting in a decrease in roughness.

### 3.4. Contact Angle Measurement

**Figure 7:**
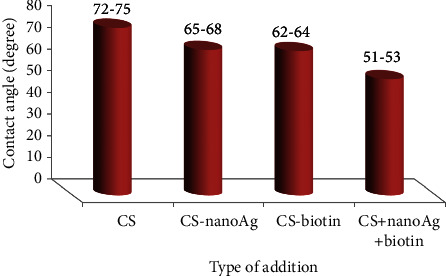
Contact angle results for CS-based composite coatings (nanosilver and biotin) on 316L SS.

The contact angle test was used to determine the surface hydrophilicity of a water droplet when it made contact with a surface. Evaluating how far a drop of water may travel over a surface is sensible, and it is an essential aspect that determines the biological reactions to implants significantly. Surface hydrophilicity is essential during the early stages of cell migration, proliferation, differentiation, and bone creation.  Figure 7 illustrates the average value of contact angles for coated specimens compared to the substrate without coating. The measurement was taken when a drop of water was deposited on the surface of the sample after 30 s.

The surface that has the smallest contact angle was for CS + nanosilver + biotin composite coating. The contact angle of this composite coating was found to be roughly 53.58 degrees because the agglomerate surface minimized the contact angle of the whole composite coating material, suggesting its hydrophilicity [50]. In comparison, other composite coatings such as those made from chitosan and silver nanoparticles have higher contact angles, indicating their hydrophobic nature [53].

**Figure 8:**
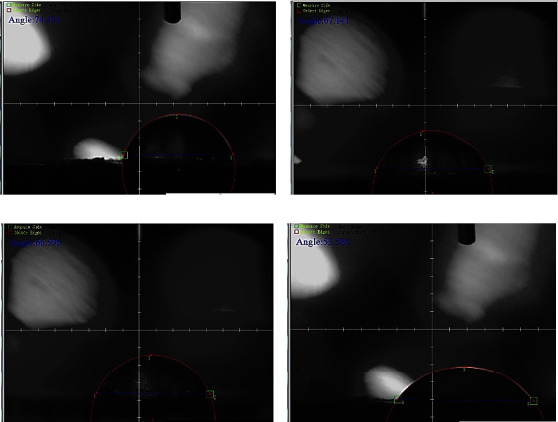
Contact angle of the (a) neat chitosan-coated 316L SS substrate, (b) nanosilver in CS-based composite-coated 316L SS substrate, (c) biotin in CS-based composite-coated 316L SS substrate, and (d) biotin and nanosilver in CS-based composite-coated 316L SS substrate.

Figure 8(a) shows the hydrophilic surface of the neat CS coating which gives a contact angle of 75.29 degrees. Incorporating nanosilver through chitosan increased the hydrophilicity (see Figure 8(b)) which gives a contact angle of about 65.98 degrees. The hydrophilicity also increased when adding biotin to the CS matrix when the contact angle reached 63.2 degrees (see Figure 8(c)), indicating that the surface's hydrophilicity increased after the incorporation of nanoparticles. In spite of the decreased roughness and increased surface heterogeneity due to nanosilver and biotin additions, the drops in all samples were nearly entirely dispersed and were absorbed by the composite coating, which demonstrates a good wettability due to the presence of nanoparticles. Finally, the superior hydrophilic surface with a contact angle of 53.58 degrees (see Figure 8(d)) was for CS + nanosilver + biotin coated sample. It can be considered the best hydrophilic coating in this investigation [54].

## 4. Conclusions

Through this study, it had been aimed to improve the surface characteristics of 316L stainless steel for biomedical applications by coating it using dip-coating technique. Chitosan-based composites enhanced with nanosilver and biotin materials were utilized as different coatings depending on the biocompatibility property of these materials to enhance the interaction degree between 316L stainless steel and cells. The SEM images revealed that the coating layer had a uniform distribution of nanosilver and biotin in the chitosan CS polymer matrix even though it was aggregated. This outcome achieved the proposed technique's applicability. Furthermore, the addition of both materials considerably decrements the intensity of the FTIR peaks of chitosan. The hydrophilicity of the implant surface was enhanced with the decreasing contact angle when adding biotin or nanosilver, and the better hydrophilic property with a contact angle of 53.58 degrees is obtained by adding both nanosilver and biotin. AFM images reveal the good incorporation of nanosilver and biotin within chitosan structure. So, all these results make these composite coating materials a good candidate for biomedical applications.

## Figures and Tables

**Table 1:** Coating composition.

Sample no.	Coating composition wt.%
1	100% neat chitosan
2	90% chitosan + 10% nanosilver (silver was preprepared by Abed et al. [39])
3	90% chitosan + 10% biotin
4	90% chitosan + 5% nanosilver + 5% biotin

**Table 2:** Surface roughness and average diameter of chitosan base composite coating.

Type of substrate	Type of mix (wt %)	Surface roughness (nm)	Average dia. (nm)
316L SS	Chitosan	12	115.18
	CS + nanoAg	8.68	66.70
	CS + biotin	4.21	95.22
	CS + nanoAg + biotin	3.33	76.72

## Data Availability

The data utilized and analyzed during this investigation are available.
